# The golden key to open mystery boxes of SMARCA4-deficient undifferentiated thoracic tumor: focusing immunotherapy, tumor microenvironment and epigenetic regulation

**DOI:** 10.1038/s41417-024-00732-4

**Published:** 2024-02-13

**Authors:** Xiang Li, Sen Tian, Hui Shi, Na Ta, Xiang Ni, Chenguang Bai, Zhanli Zhu, Yilin Chen, Dongchen Shi, Haidong Huang, Longpei Chen, Zhenhong Hu, Lei Qu, Yao Fang, Chong Bai

**Affiliations:** 1 Department of Respiratory and Critical Care Medicine, the First Affiliated Hospital of Naval Medical University (Shanghai Changhai Hospital), Shanghai, China; 2 Department of Respiratory and Critical Care Medicine, General Hospital of Central Theater Command of the Chinese People’s Liberation Army, Wuhan, China; 3 Department of Respiratory and Critical Care Medicine, No. 906 Hospital of the Chinese People’s Liberation Army Joint Logistic Support Force, Ningbo, China; 4 Department of Pathology, the First Affiliated Hospital of Naval Medical University (Shanghai Changhai Hospital), Shanghai, China; 5 Department of Oncology, the First Affiliated Hospital of Naval Medical University (Shanghai Changhai Hospital), Shanghai, China

**Keywords:** Non-small-cell lung cancer, Cancer microenvironment, Cancer immunotherapy

## Abstract

SMARCA4-deficient undifferentiated thoracic tumor is extremely invasive. This tumor with poor prognosis is easily confused with SMARCA4-deficent non-small cell lung cancer or sarcoma. Standard and efficient treatment has not been established. In this review, we summarized the etiology, pathogenesis and diagnosis, reviewed current and proposed innovative strategies for treatment and improving prognosis. Immunotherapy, targeting tumor microenvironment and epigenetic regulator have improved the prognosis of cancer patients. We summarized clinicopathological features and immunotherapy strategies and analyzed the progression-free survival (PFS) and overall survival (OS) of patients with SMARCA4-UT who received immune checkpoint inhibitors (ICIs). In addition, we proposed the feasibility of epigenetic regulation in the treatment of SMARCA4-UT. To our knowledge, this is the first review that aims to explore innovative strategies for targeting tumor microenvironment and epigenetic regulation and identify potential benefit population for immunotherapy to improve the prognosis.

## Introduction

SMARCA4-deficient undifferentiated thoracic tumor (SMARCA4-UT) is characterised by inactivating mutation of *SMARCA4* gene along with undifferentiated or rhabdoid morphology. Nonsense mutations are the most common, followed by frameshift mutations. Splicing mutations and missense mutations can likewise inactivate *SMARCA4* gene [1–3]. SMARCA4-UT shares similar morphological and transcriptomic features with malignant rhabdoid tumor (MRT) and small cell carcinoma of the ovary-hypercalcemic type (SCCOHT), but without germline mutations [1]. It is closely associated with tobacco exposure and is frequently accompanied by *TP53* mutations [1, 3, 4]. Moreover, smoking-related non-small cell lung cancer (NSCLC) mutations, including *STK11*, *KEAP1* and *KRAS*, are not uncommon [1, 3, 4]. Therefore, it was once proposed as a new type of thoracic tumor and named SMARCA4-deficient thoracic sarcoma (SMARCA4-DTS) [1]. However, SMARCA4-DTS represents smoking-associated undifferentiated/de-differentiated carcinoma with unique clinicopathologic characteristics [3]. The World Health Organization (WHO) has classified SMARCA4-DTS as “other epithelial tumors of the lung” and renamed it as SMARCA4-UT [5].

Male is overwhelmingly dominant in SMARCA4-UT [1, 2, 6, 7] and it commonly occurs in young adults. The median age is 48 years old (range from 28 to 90 years) [8]. SMARCA4-UT is extremely invasive with poor efficacy and high mortality. Metastatic diseases account for 77% to 83% [2, 6, 8]. The median overall survival (mOS) is 6 months [8]. Until the year of 2021, the number of reported cases was less than 100 cases [9]. However, SMARCA4-UT has received increasing attention since 2021. In order to improve the diagnosis and prognosis of patients with SMARCA4-UT, this article reviewed the diagnostic and therapeutic strategies, particularly immunotherapy, tumor microenvironment (TME), epigenetic regulation and novel targeted therapy.

## Etiology and pathogenesis

### Etiology

Although the exact etiology is still unclear, there is evidence that most patients are smokers or ex-smokers [1–3, 7] and the proportion of patients who have a history of smoking is up to 87% [8].

### Pathogenesis

**Fig. 1:**
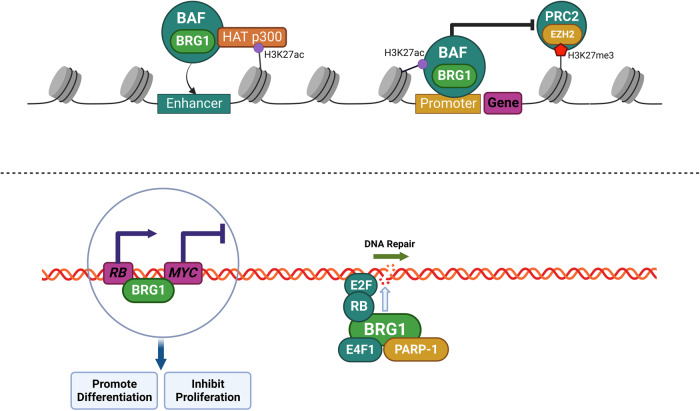
The role of BAF complexes and Polycomb Group (PcG) protein. By binding to *MYC* or *MYC* promoters, BRG1 represses *MYC* expression, thereby promoting cell differentiation and inhibiting cell proliferation. E4F1 binds to BRG1 and PARP-1, followed by gathering at DNA damage sites and promoting DNA repair. BAF complexes are recruited to promoters of E2F targeted gene by RB and E2F, and promote DNA damage repair.(Created with BioRender.com).

The SWI/SNF family of chromatin-remodelling complexes, also known as BRG1/BRM-associated factor (BAF) complexes in human, consist of specific subunits and are classified as canonical BAF (cBAF), polybromo-associated BAF (PBAF), and non-canonical BAF (ncBAF). The core of all three complexes is composed of SMARCC, SMARCD and either SMARCA4 (also known as BRG1) or SMARCA2 (also known as BRM) (Table 1) [10, 11]. The BAF complexes depend on the ATPase activity of BRG1 or BRM to promote nucleosome dissociation and chromosome remodeling and play critical roles in transcription, differentiation, and DNA repair [10]. The BAF complexes interact with histone acetyltransferase (HAT) p300, upregulating histone H3 lysine 27 acetylation (H3K27ac) on the targeted genes and promoting BAF complexes to bind to enhancers [12], and the BAF complexes evict Polycomb repressor complexes (PRC2, one core protein of PcG complex) in an ATP-dependent manner [13]. However, enhancer of zeste homologue 2 (EZH2, enzymatic subunit of PRC2) trimethylates histone H3K27 to antagonize the activity of BAF complexes (Fig. 1) [14].

**Table 1:** Components of BAF complexes in human.

BAF complex	Shared subunits	Specific subunits
cBAF	SMARCC1/2, SMARCD1/2/3, SMARCA4/2, β-Actin, ACTL6, BCL7A/B/C	SMARCB1, SMARCE1, SS18/L1, ARID1A/B, DPF1/2/3
PBAF	SMARCC1/2, SMARCD1/2/3, SMARCA4/2, β-Actin, ACTL6, BCL7A/B/C	SMARCB1, SMARCE1, ARID2, PBRM1, BRD7, PHF10
ncBAF	SMARCC1/2, SMARCD1/2/3, SMARCA4/2, β-Actin, ACTL6, BCL7A/B/C	SS18/L1, BRD9, GLTSCR1/1L

Following SMARCA4 deficiency, *MYC* gene activation expression [15] and the termination of cell cycle arrest which is mediated by *RB* gene [16] lead to inhibit differentiation and promote proliferation. Moreover, E4F transcription factor 1 (E4F1) and poly ADP-ribose polymerase-1 (PARP-1) are unable to gather at DNA damage sites and perform DNA repair [17], and the initiation of *RB* gene mediated DNA damage repair is inaccessible (Fig. 1) [18].

## Clinical manifestation

Patients with SMARCA4-UT often presented with huge masses in thorax, including mediastinum, lungs or pleura, and clinical manifestations are different from those of lung cancer. Dyspnea, chest pain or superior vena cava syndrome caused by compression of huge mediastinum masses or lung masses were prominent and common presentations [7]. Patients also presented with cough, Pancoast syndrome, hemoptysis, dysphagia, abdominal pain, and digestive hemorrhage [1, 6, 7]. Furthermore, symptoms of brain or bone metastases such as neurologic disorders, aphasia and difficulty in writing, or ostalgia also accounted for patients visits [6, 19, 20]. These depend on tumor location, size, metastases and complications.

## Imaging characteristics

**Fig. 2:**
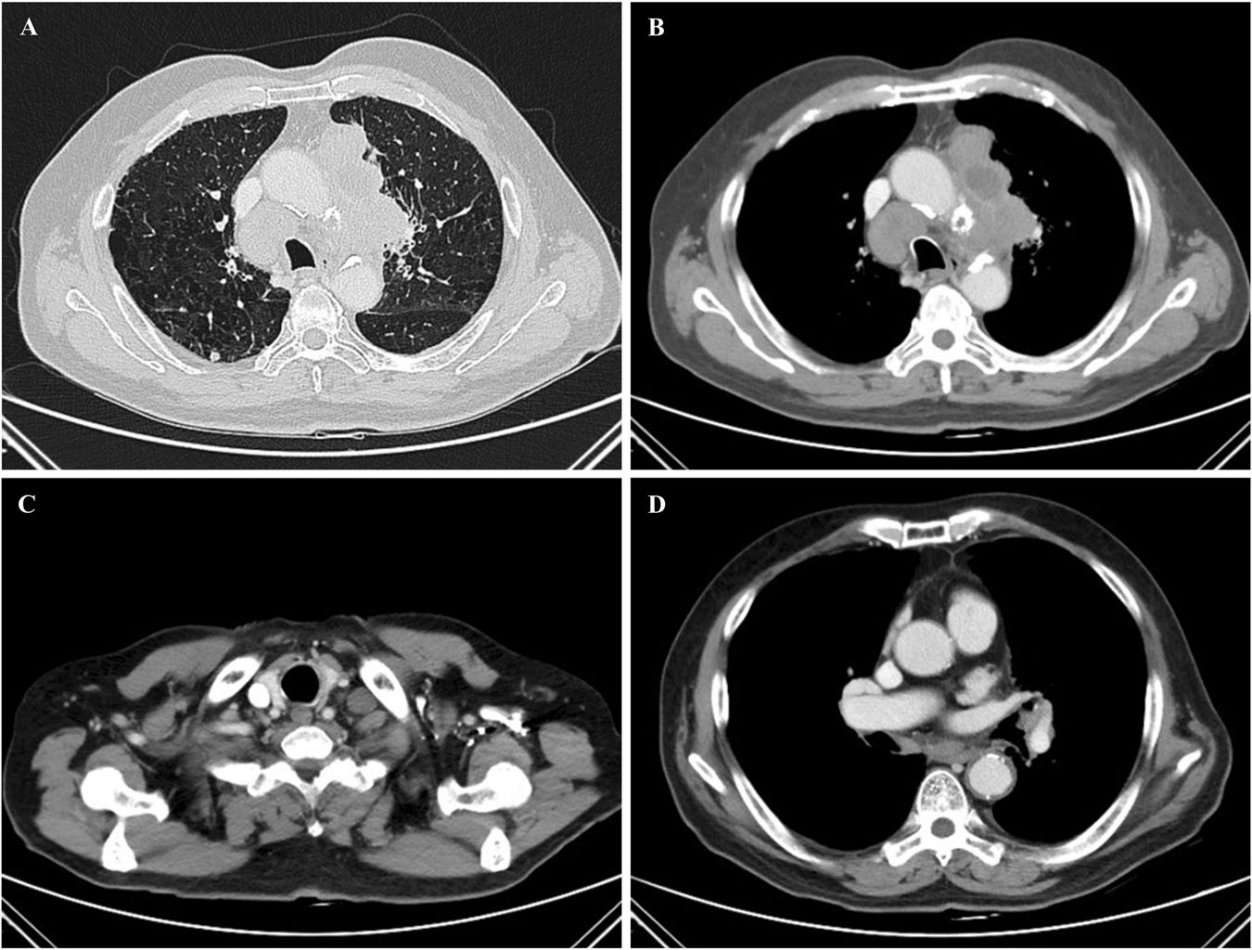
Imaging characteristics of SMARCA4-UT. **A** Plain chest CT scanning illustrated the huge mass which involved left upper lobe and mediastinum, and bilateral emphysema and pulmonary bullae. **B** Enhanced chest CT scanning showed that the mass was heterogeneous enhanced and mediastinal lymphadenopathy. **C**, **D** Supraclavicular and subcarinal lymphadenopathy.

The chest plain CT scans showed that huge invasive masses were in the thorax, which was a prominent finding of patients with SMARCA4-UT. Tumors frequently originated from mediastinum, lung or pleura. The median and maximum diameter were 130 mm and 266 mm, respectively. Lung emphysema was present on chest CT of more than 50% of patients (Fig. 2) [1, 7]. When tumors originated from or involved the pleura, multiple pleural lesions or one huge mass were both unilateral and often accompanied by ipsilateral pleural effusion [7]. Enhanced chest CT scans showed ill-defined and heterogeneous enhanced masses without calcification or cystic component (Fig. 2). Tumors often compressed the airway leading to atelectasis, surrounded mediastinal vessels, and compressed or invaded esophagus and chest wall [1, 7, 8].

Mediastinal, intrathoracic and cervical lymphadenopathy were frequent (Fig. 2). Intraperitoneal, retroperitoneal or axillary lymphadenopathy were present in approximately one quarter of patients. Furthermore, the involved lymph nodes were often ill-defined with necrosis and surrounding fatty infiltration [6, 7].

About 80% of patients with SMARCA4-UT presented with metastatic diseases, and extrathoracic metastatic sites were predominantly bone and adrenal gland. Liver, digestive tract, brain and kidney involvement have also been reported [2, 3, 6–8]. Positron Emission Tomography/ Computed Tomography (PET/CT) contributes to detecting metastases and tumor stage. The 18F-flurodeoxyglucose (18F-FDG) of the lesion was often highly concentrated, and maximum standard uptake value (SUVmax) was 7 to 33.8 [3, 7].

## Diagnosis and pathology

Although smoking is strongly associated with SMARCA4-UT and imaging findings have certain prompting implications, SMARCA4-UT cannot be diagnosed based on them alone. Likewise, neither clinical manifestations nor laboratory tests are specific. Therefore, the diagnosis ultimately remains dependent on pathology.

### Histology

**Fig. 3:**
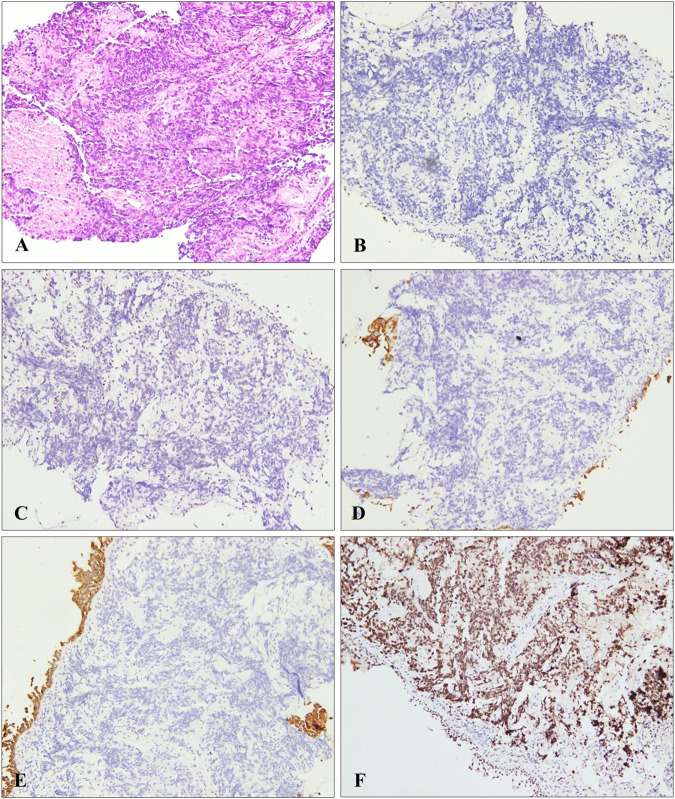
Pathology of SMARCA4-UT. **A** H&E staining: In tumor, undifferentiated round or spindle shaped tumor cells of varying sizes with prominent nucleoli were arranged around vessels, with focal rhabdoid features and sheet necrosis. SMARCA4 and SMARCA2 expression of tumor cells was absent (**B** and **C**, respectively) (Note endothelial and inflammatory cells as internal positive controls). Immunohistochemistry staining was negative for CK-7 and Pan-cytokeratin in tumor cells (**D** and **E**, respectively). Diffuse expression of SOX2 in tumor cells (F).

SMRACA4-UT was undifferentiated or poorly differentiated neoplasm [2, 7, 9]. Tumor cells were relatively monotonous and could be epithelioid, round or rhabdoid [2, 3, 6]. They were arranged in sheets or nests with massive necrosis and often infiltrated surrounding tissues [1–3, 7]. Cytoplasm was moderate to abundant, eosinophilic or clear. The nucleus was large and the nucleolus was prominent (Fig. 3) [1, 2, 6]. Meanwhile, mitosis was active [1, 3, 6].

### Immunohistochemistry

The histological morphology of SMARCA4-UT was not significantly characteristic and therefore immunohistochemical (IHC) staining should be performed to confirm the diagnosis in suspected cases.

Expression of SMARCA4 was absent, and that of SMARCA2 was absent in the vast majority of cases (Fig. 3) [3, 5], while that of SMARCB1 was retained [1–3] and immunohistochemical staining was frequently positive for stem cell markers including SOX2, CD34 or SALL4 (Fig. 3) [1, 2, 7, 9].

For pan-cytokeratin (including AE1/AE3 or KL1) [1] and keratin (including CAM5.2, CK5/6 or CK7) (Fig. 3) [1, 3, 6], immunohistochemical staining was usually negative or focally positive. However, immunohistochemical staining for EMA was variable [1, 3] and was negative or only locally positive for Claudin-4 [1–3].

Most cases were negative for TTF-1 or p63/p40, and few positive cases were focal [1, 3, 6, 7]. Moreover, immunohistochemical staining for Napsin A and chromogranin A was commonly negative, and that for synaptophysin was variable [1–3, 7]. The sensitivity and specificity of SMARCA4-UT immunohistochemical signature were 87.5% and 99.5%, respectively [8].

### Molecular pathology

Sanger sequencing (Sanger-seq) [1, 21], fluorescence in situ hybridization (FISH) [3], and next-generation sequencing (NGS) including RNA sequencing (RNA-seq) [1, 7] and targeted NGS [2, 22] have been utilised to diagnosis SMARCA4-UT. Sanger-seq is a highly accurate and low sequencing throughput first-generation sequencing technology, while it is costly and time-consuming for large-scale sequencing. It is especially suitable for verifying NGS results. FISH was performed by using artificial chromosome probes targeting both sides of SMARCA4 (19p13) locus. However, SMARCA4-UT is usually copy-neutral loss of heterozygosity (CN-LOH) and only 35% of SMARCA4 alterations were detected [3]. Moreover, NGS omitted 12.5% of patients with SMARCA4-UT and immunohistochemical staining for SMARCA4 was “severe global reduction” rather than negative in few cases [3]. Therefore, the combination of IHC with NGS is recommended for the diagnosis of SMARCA4-UT.

DNA-sequencing did not detect the *SMARCA2* mutations [3, 22], while RNA-seq indicated that SMARCA2 expression was decreased [1], which suggested the loss of *SMARCA2* expression was epigenetically regulated [10]. *EGFR* mutations, *ALK* and *ROS1* rearrangement have not been reported [1, 3, 22], while frequent *TP53* mutations were detected [1, 3, 20] and the mutations of *STK11*, *KEAP1* and *KRAS* were not uncommon [1–3, 9].

## Differential diagnosis

SMARCA4-UT is an undifferentiated or poorly differentiated malignancy. Early diagnosis is extremely significant due to high aggressiveness and poor prognosis. SMARCA4 deficience occurs in approximately 10% of NSCLC [23], and most patients with SMARCA4-deficient NSCLC or SMARCA4-UT are both smoking males. It is particularly important to differentiate them. SMARCA4-UT shows undifferentiated morphology with epithelioid, round or rhabdoid tumor cells. Immunohistochemical staining is negative for SMARCA2 and Claudin-4, and is positive for SOX2 [1–3].

MRT can occur at any age, which tends to occur in infants and young children [24], and small-cell carcinoma of the ovary, hypercalcemic type (SCCOHT) is a rare malignancy origined from ovarian, which mainly occurs in adolescents and young women [25].

SMARCA4-UT, MRT and SCCOHT are morphologically similar, and MRT and SCCOHT can be likewise loss of *SMARCA2* expression [1], but the vast majority of MRT is SMARCB1-deficient [2, 24]. SMARCA4-UT has a complex genomic profile and *SMARCA4* inactivation is not present in the germline [1, 3].

Proximal-type epithelioid sarcoma overlapped with SMARCA4-UT in morphology. However, epithelioid sarcoma is SMARCB1-deficient with retained SMARCA2 expression, and the expression of SOX2 and SALL4 is deficient [2].

Both SMARCA4-UT and primary pulmonary nuclear protein of testis (NUT) carcinoma are extremely aggressive undifferentiated or poorly differentiated neoplasms. At initial visit, primary lesions are large and accompanied by lymphadenopathy and metastatic diseases. Pleural lesions are unilateral and accompanied by ipsilateral pleural effusions [26]. However, the expression of *SMARCA4* and *SMARCA2* is present and immunohistochemical staining is positive for NUT in primary pulmonary NUT carcinoma [26].

When young male patients with a heavy smoking history present with huge, undifferentiated or poorly differentiated thorax tumors, the possibility of SMARCA4-UT should be considered. Especially for tumors with obviously local infiltration accompanied by distant metastasis, poor response to chemotherapy and rapidly local progression, immunohistochemical staining for SMARCA4, SMARCA2, SOX2 and Claudin-4 should be performed in combination with NGS that can detect gene alterations.

## Therapy strategies

No standard and efficient treatment for SMARCA4-UT has been established so far [20, 24]. Since SMARCA4-UT is obviously local invasiveness and is usually associated with distant metastases, it is determined that the treatment is mainly non-surgical, mainly including traditional chemotherapy and radiotherapy, and immunotherapy and anti-angiogenesis therapy that have attracted much attention in recent years. Moreover, targeted therapy is also the focus in current cancer research. Due to low incidence of SMARCA4-UT, which was once defined as a new type of sarcoma, and low detection rate of driver genes, nearly no positive cases of driver genes have been reported so far, so there are few clinical applications. Novel targeted agents may be potential treatments.

### Surgery

Radical surgery is undoubtedly the preferred therapy for solid malignant tumors. However, SMARCA4-UT is prone to local invasion and distant metastasis. Therefore, only a minority of patients with SMARCA4-UT received surgical resection.

For resectable tumors, patients appeared to benefit from first-line surgery (or adjuvant chemotherapy and even combined with Bevacizumab concurrently), and the OS was prolonged (the longest OS was 20 months, patient 1 and 2 in Supplementary Table 1) [1]. However, patients did not seem to significantly benefit from first-line neoadjuvant therapy and surgery, and the best OS was only 11 months (patient 3 and 4 in Supplementary Table 1) [1].

Although the OS was prolonged in some patients who underwent surgery in the first-line, the disease progressed rapidly after surgery. Disease progression in SMARCA4-UT was dominated by local invasion [1], and the disease was stable for 3 months for one stage IIA patient who underwent surgery and adjuvant chemotherapy in the second-line after palliation therapy in the first-line (patient 5 in Supplementary Table 1) [22]. Therefore, for patients who previously underwent surgery, it remains possible that the patient may benefit from surgery after disease progression.

In conclusion, patients with early or locally advanced (IIIA stage without lymphadenopathy) disease can be treated with radical surgery and adjuvant chemotherapy (or combined with Bevacizumab concurrently) in the first-line. For patients who have undergone surgery and locally progressed, reoperation remains significant.

### Chemoradiotherapy and chemotherapy

Patients with unresectable or inoperable SMARCA4-UT have commonly undergone chemotherapy or chemoradiotherapy. At present, it is considered that SMARCA4-UT has a poor response to chemotherapy [5, 20]. For unresectable or inoperable stage III disease, the best PFS of patients who received doxorubicin monotherapy in the first-line was 20 weeks (patient 6 in Supplementary Table 1) [21], while the OS of patients who underwent chemotherapy was only 2 months to 7 months. For stage IIIB-IV disease, the best PFS of patients who received doxorubicin monotherapy or etoposide combined with cisplatin in the first-line was 18 weeks, while the OS of these patients was less than 9 months (patient 7 and 8 in Supplementary Table 1) [1]. Furthermore, a considerable number of patients did not respond to one or more chemotherapy regimens and the OS was less than half a year (the shortest OS was 1 month) [1, 6, 19]. However, Bell et al. have reported that low expression of *SMARCA4* could predict increased sensitivity to platinum-based chemotherapy in lung cancer [27]. Although the best PFS of patients with stage IV disease who underwent chemoradiotherapy was only 9 weeks, the OS usually exceeded 9 months, and even a patient had survived for more than 12 months (patient 9 in Supplementary Table 1) [1]. Compared with chemotherapy alone, chemoradiotherapy seemed to improve the prognosis.

### Targeted therapy

So far, only one case with *MET* amplification was reported, while classic driver oncogenes including *EGFR* mutations, *ALK* and *ROS1* rearrangements in lung cancer have not been reported [1]. Although the PFS of one patient (stage III) without *EGFR* mutation who underwent erlotinib in the second-line after surgery in the first-line was 15 months and the OS was 20 months (patient 1 in Supplementary Table 1) [1], the significance of traditional targeted therapy in SMARCA4-UT remains unclear. In recent years, novel targeted therapy is gradually emerging.

#### Cyclin Dependent Kinases (CDK) 4/6 inhibitor

Cyclin D1 expression decreased and the sensitivity to CDK4/6 inhibitor increased in tumors with the loss of SMARCA4 or SMARCA2 [28]. The inhibitory effect of CDK4/6 inhibitor on tumor growth has been confirmed in lung cancer [28, 29]. Abemaciclib has already shown extraordinary promise in advanced breast cancer [30], and it is being applied for the indication for lung cancer.

#### Oxidative Phosphorylation (OXPHOS) inhibitor

OXPHOS was increased in tumors with *SMARCA4* mutation and OXPHOS inhibitor (IACS-010759) represses OXPHOS, thereby inducing tumor cell death and inhibiting tumor growth [31]. Phase 1 trial in solid tumors has demonstrated that 44% of patients who received IACS-010759 acquired stable disease (SD) or partial response (PR) [32].

#### KRAS inhibitor

**Fig. 4:**
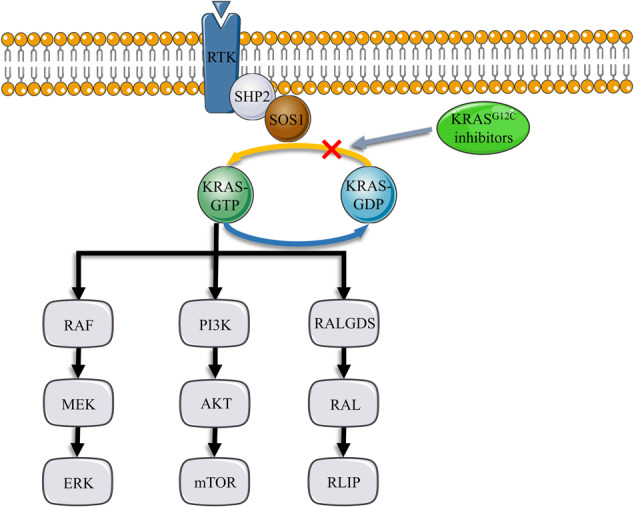
KRAS pathway and KRAS inhibitors. KRAS^G12C^ inhibitors selectively and irreversibly binds KRAS^G12C^ in its inactive GDP state.

Patients with *KRAS*-mutant NSCLC had a poor prognosis [33], and *KRAS*^*G12C*^ mutation with a poorer prognosis compared with other *KRAS* mutations [34] accounted for 42% of *KRAS*-mutant lung adenocarcinoma [35]. KRAS^G12C^ inhibitors those selectively and irreversibly binds KRAS^G12C^ in its inactive GDP state (Fig. 4) have been emerging in recent years [36]. Sotorasib made a durable clinical benefit which an objective response and disease control occurred in 37.1% and 80.5% of patients with previously treated *KRAS*-mutant NSCLC, and median PFS (mPFS) and mOS was 6.8 and 12.5 months, respectively [37]. Subsequently, a randomised, phase 3 trial demonstrated that Sotorasib significantly increased PFS for previously treated *KRAS*-mutant NSCLC [38]. Adagrasib made an objective response in 42.9% of patients with previously treated *KRAS*-mutant NSCLC, and mPFS and mOS was 6.5 and 12.6 months, respectively [39]. Garsorasib seemed to achieve comparable results [40], and Divarasib was more promising with a disease response rate of 53.4% and mPFS of 13.1 months [41]. Rekhtman [3] reported that *KRAS* mutation occurred in 27.8% of patients with SMARCA4-UT. KRAS inhibitor combination therapy may benefit patients accompanied by *KRAS* mutations.

#### Targeting KEAP1 and Ataxia-telangiectasia-mutated (ATM) kinase

Li [42] identified ATM and KEAP1 as new targets in lung cancer, and KEAP1 deficiency sensitized lung tumors to ATM inhibition. Novel ATM inhibitors are currently under phase I clinical trials [42]. ATM and KEAP1 dual-targeted therapy may be beneficial.

#### AXL inhibitor

AXL is a receptor tyrosine kinase that is often overexpressed in cancers [43]. Bemcentinib restored pembrolizumab sensitivity of *STK11/LKB1* mutant NSCLC through expansion of PD-1^+^ CD8^+^ T cells [44], and it has been granted fast track designation by the U.S. Food and Drug Administration (FDA) in *STK11* mutated advanced metastatic NSCLC [43].

### Epigenetic regulation

#### Bromodomain and Extra-Terminal Domain Protein Inhibitor (BETi)

BET binds to histone acetylated lysine residues in chromatin, thereby promoting tumorigenesis and tumor proliferation [45], and BETi has been demonstrated as an antitumor drug for lung cancer [46, 47]. In addition, BETi increased the sensitivity of tumor cells to CD8^+^ T cells, and enhanced tumor growth inhibition in a TNF-dependent manner [48]. In SMARCA4/A2-deficient lung cancer model, BETi significantly repressed tumor growth [49]. However, *KRAS*-mutant lung cancer was resistant to BETi [50], and it may limit BETi monotherapy in SMARCA4-UT which is usually accompanied by *KRAS* mutation.

#### Aurora Kinase A (AURKA) Inhibitor

In SMARCA4-deficient tumor cells, the activity of AURKA is necessary for mitotic spindle assembly and cell survival, and it has been demonstrated that the AURKA inhibitor (VX-680) induces tumror cells death in vitro and in mouse vivo assays [51]. Shi [52] found that AURKA was a potential lung cancer marker by KEGG and GO enrichment analysis, and Alisertib has shown promising clinical activity in solid tumors [53].

#### Inhibitors of DNA damage repair

E4F1 is dependent on PARP to be recruited to DNA damage sites and promote DNA repair, and PARP inhibitor impairs DNA damage repair, thereby cell death [17]. PARP inhibitor in combination with radiotherapy showed a synergistic effect in the treatment of SWI/SNF mutant tumors [54], and sensitized lung cancer to PD-1 inhibitor immunotherapy [55]. In addition, Niraparib maintenance therapy modestly improved the PFS of lung cancer patients [56]. Ataxia-Telangiectasia Mutated and Rad3-related protein kinase (ATR) is a DNA damage checkpoint kinase and ATR inhibitor can trigger genomic instability and apoptosis in SWI/SNF mutant tumors [57].

#### Histone Deacetylase Inhibitor (HDACi)

HDACi can restore *SMARCA2* expression in a variety of SMARCA2-deficient cell lines, thereby inhibiting tumor cells proliferation [58]. Furthermore, HDACi can recover inositol 1,4,5-trisphosphate receptor type 3 (IP3R3) expression and enhance cisplatin sensitivity in SMARCA4/A2-deficent tumor cells [59]. HDACi monotherapy does not appear to be satisfactory in solid tumors [60]. In recent years, HDACi has been increasingly used in immunoregulation or combination therapy. HDACi enhanced the expression of costimulatory molecules, promoted tumor neoantigen presentation, the migration and infiltration of CD8^+^ T cells into tumors and M1 polarization of macrophages, increased antitumor efficacy of immune checkpoint inhibitor (ICI) [61, 62], and decreased regulatory T cells in TME [63]. In clinical practice, early clinical studies confirmed that combination of vorinostat with pembrolizumab enhanced and restored sensitivity to PD‐1 inhibitor [64], and Pembrolizumab combined with vorinostat has demonstrated preliminary antitumor activity in NSCLC previously treated with ICIs [65]. HDACi restored SMARCA2 expression, enhanced cisplatin sensitivity and restored sensitivity to PD‐1 inhibitor. We proposed that patients with SMARCA4-UT may benefit from combination therapy of HDACi, cisplatin-based chemotherapy and PD-1 inhibitor.

### Immunotherapy

#### The present condition of immunotherapy in SMARCA4-UT

For stage IV patients with PD-L1 expression less than 1%, there was no response to nivolumab monotherapy or ipilimumab in combination with nivolumab in the first-line, and the OS was less than 3 months (patient 10 and 11 in Supplementary Table 1) [19]. The OS of one patient who underwent carboplatin in combination with paclitaxel in the first-line and nivolumab in the second-line was only 6.5 months (patient 12 in Supplementary Table 1) [19]. After receiving chemoradiotherapy (carboplatin combined with paclitaxel) in the first-line, patients with stage IVB who underwent nivolumab or pembrolizumab monotherapy in the second-line or beyond treatment can acquire partial response (PR) and the OS was significantly prolonged (The best survival duration was 22 months and the patient was alive) (patient 13 and 14 in Supplementary Table 1) [66, 67]. Moreover, the PFS and OS of a patient with stage IVA who received carboplatin combined with paclitaxel plus immune checkpoint inhibitor in the first-line was 24 weeks and 11 months, respectively (patient 15 in Supplementary Table 1) [4].

One stage IV patient with PD-L1 expression of 10% who underwent permetrexed in combination with carboplatin plus pembrolizumab in the first-line, acquired PR after 3 months of initial treatment, and the patient has survived for 11 months with stable disease (patient 16 in Supplementary Table 1) [68].

For stage IVB patients with at least 50% PD-L1 expression, the PFS of those underwent pembrolizumab monotherapy in the first-line was 24 weeks and the OS was significantly prolonged (The best OS was 26 months) (patient 17 and 18 in Supplementary Table 1) [69, 70].

#### The recommendations for the application of immunotherapy in SMARCA4-UT

Therefore, for unresectable tumors or inoperable patients, when PD-L1 expression is at least 50%, they are likely to benefit more from Pembrolizumab or Nivolumab (especially Pembrolizumab) monotherapy in the first-line. Chemotherapy combined with Pembrolizumab in the first-line seems to be associated with better prognosis for patients with PD-L1 expression of at least 1%. For patients with PD-L1 expression less than 1%, chemoradiotherapy (carboplatin combined with paclitaxel) in the first-line and nivolumab or pembrolizumab monotherapy in the second-line or beyond treatment could improve the prognosis.

**Fig. 5:**
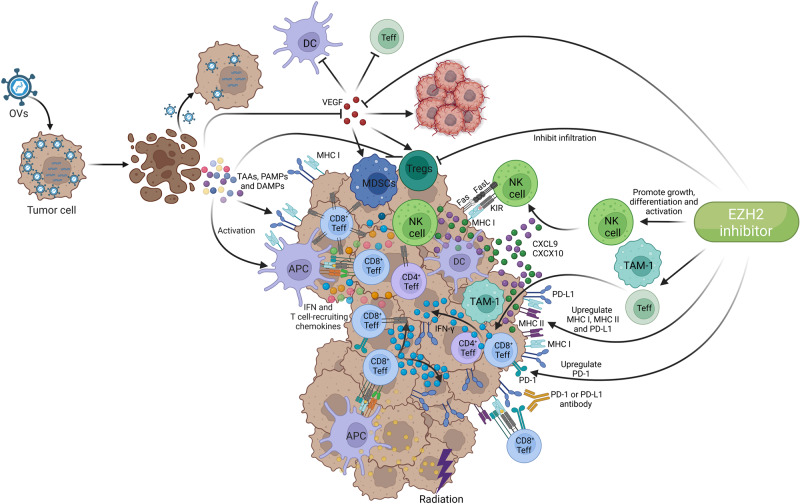
The main mechanisms of therapy targeting TME in SMARCA4-UT. Tumor-associated antigens (TAAs), those released by tumor cells killed by radiation, are presented to immune cells, and activated CD8^+^ Teffs are recruited to the tumor and release IFN-γ. The level of PD-1 on CD8^+^ Teffs and PD-L1 on tumor cells are increased, and tumor cells can be killed accompanied by enhanced response of PD-1 inhibitors. VEGF inhibits DCs maturation and the activation and infiltration of Teffs, while upregulating the activity of MDSCs and Tregs. OVs selectively kill cancer cells, thereby releasing TAAs, PAMPs and DAMPs to activate APCs. OVs promote the production of IFN and T cell-recruiting chemokines to recruit Teffs into the tumor, and induce PD-L1 expression on tumor cells and MHC I expression. Furthermore, oncolytic virotherapy decreases immunosuppressive cells, and reduces VEGF levels in TME. EZH2 inhibitor promotes the growth, differentiation and activation of NK cells, and promotes the secretion of CXCL9 and CXCL10 to recruit NK cells, M1 TAMs, and CD4^+^ and CD8^+^ Teffs to the tumor, thereby killing tumor cells. EZH2 inhibitor upregulates the expression of MHC I and MHC II and increases the expression of PD-L1 on tumor cells and PD-1 on CD8^+^ Teffs. Furthermore, EZH2 inhibitor decreases Tregs infiltration and inhibits VEGF-A/AKT signaling pathway.(Created with BioRender.com).

The OS of patients with PD-L1 expression less than 1% who underwent chemoradiotherapy in the first-line and ICI in the second-line are comparable to that of patients with PD-L1 expression at least 50% who received pembrolizumab monotherapy in the first-line. The rationale was described in our previous article [26] and was shown in Fig. 5.

PD-1 inhibitor, especially Pembrolizumab, in combination with HDACi, KRAS^G12C^ inhibitor in patients with *KRAS*^*G12C*^ mutation (or KRAS^G12C^ inhibitor monotherapy as second-line or beyond therapy), or AXL inhibitor in patients with *STK11/LKB1* mutation, may be treatment options.

## Targeting the tumor microenvironment

### Anti-vascular combined immunotherapy modulates the tumor microenvironment

Bevacizumab promotes dendritic cell maturation, and the activation and infiltration of T cells while downregulating the activity of myeloid-derived suppressor cells (MDSCs) and regulatory T cell (Treg) and normalizing the tumor vasculature [26] (Fig. 5).

According to IMpower150 research, atezolizumab plus bevacizumab plus carboplatin and paclitaxel (ABCP) improved the PFS and OS. The patients with SMARCA4-UT have undergone ABCP [20]. One stage IVB patient without PD-L1 expression, the PFS reached 10 months and the patient lived for at least 10 months (patient 19 in Supplementary Table 1) [20]. One stage IVA patient with 40% of PD-L1 expression acquired PR after 3 cycles and had a stable disease, and lived for at least 17 months (patient 20 in Supplementary Table 1) [20]. However, the PFS of a stage IVA patient with 80% of PD-L1 expression was just 12 weeks (patient 21 in Supplementary Table 1) [20], which may be related to *KEAP1* mutation which is a negative predictor of immunotherapy response and prognosis [71]. Therefore, patients without *KEAP1* mutation may achieve rapid response to ABCP and best PFS.

### Tumor microenvironment of SMARCA4-UT

Gantzer et al. [19]. assessed tertiary lymphoid structures (TLS), immune cell markers and immune checkpoints using immunostaining, revealed that SMARCA4-UT was mainly immune desert phenotype. The patient with TLS in tumor benefit from comprehensive therapy of surgery and ICIs, survival duration was almost 2 years (patient 22 in Supplementary Table 1) [19]. Therefore, TLS may be a potential biomarker to predict the benefit from ICIs therapy.

Oncolytic viruses (OVs) selectively replicated in and killed cancer cells [72, 73], thereby releasing tumor associated antigens (TAAs), pathogen-associated molecular patterns (PAMPs) and danger-associated molecular patterns (DAMPs) and activating APCs [73], and simultaneously induced higher expression of major histocompatibility complex (MHC) class I (Fig. 5) [74]. Moreover, OVs infected and activated APCs without causing APCs lysis, promoting the production of type I interferon and T cell-recruiting chemokines [73, 75], and subsequently recruiting T cells into the tumor [73, 76], and finally exerted an antitumor T cells response (Fig. 5) [73]. Furthermore, oncolytic virotherapy decreased immunosuppressive cells [74] and reduced vascular endothelial growth factor (VEGF) levels in TME [76]. In mice, intravenous oncolytic virus replicated in tumor-associated vascular endothelial cells and caused tumor necrosis with unaffected normal vessels (Fig. 5) [77].

In melanoma, OVs therapy increased the effector T cell (Teff), Teff to Treg ratio, memory T cell, PD-L1 expression and IFN-γ expression in tumors and circulating CD8^+^ and CD4^+^ T cells in peripheral blood [78]. Phase II trial demonstrated that compared with immunotherapy monotherapy, OVs therapy plus immunotherapy significantly improved objective response rate (ORR) and durable response rate (DDR) for advanced melanoma [79].

### EZH2 and tumor microenvironment

#### Reasons to focus on EZH2 and tumor microenvironment

Due to the inhibition of Polycomb group protein (PcG) by BAF complex [80], following SMARCA4 and/or SMARCA2 loss, EZH2 activity enhanced, oncogene activated, and tumor suppressor gene suppressed [81].

There is evidence that PcG and SWI/SNF are mutual antagonists [80]. SMARCA4 or/and SMARCA2 deficiency result in the deletion of SWI/SNF function and abnormal EZH2 activity [80, 82].

EZH2 overexpression is associated with rapid tumor progression [83] and EZH2 plays a critical role in the immune system [84].

#### The effect of EZH2 inhibition on innate immunity

NK cell-mediated killing of tumor cells is the first barrier to tumor immunity. EZH2 inhibition promoted the growth, differentiation and activation of NK cells, and then increased the antitumor activity (Fig. 5) [85]. At the same time, it promoted the secretion of CXCL9 and CXCL10 to recruit NK cells to the tumor [86], killing tumor cells (Fig. 5) [87]. Moreover, EZH2 inhibitor can reverse the inhibition of the infiltration of M1 TAM in the TME by EZH2 (Fig. 5) [88, 89].

#### The effect of EZH2 inhibition on adaptive immunity

##### EZH2 inhibition enhanced antigen processing and presentation

Major histocompatibility complex (MHC) I and MHC II are the critical molecules for antigen presentation. EZH2 mediated the expression suppression of MHC I and MHC II, while EZH2 inhibition increased tumor immunogenicity, the expression of MHC I and MHC II, and the function of antigen presentation, thereby promoting CD8^+^ T cell-mediated tumor cell killing and increasing the inhibitory effect of PD-1 or PD-L1 inhibitors on tumor growth (Fig. 5) [90–93].

##### The effect of EZH2 inhibition on regulatory T cells (Tregs)

EZH2 contributed to stabilizing the functional phenotype of activated Tregs [94]. EZH2 expression was increased in Tregs from patients who were treated with ipilimumab [95], while EZH2 inhibitor decreased Tregs stability, inhibited Tregs infiltration, attenuated the inhibitory activity of intratumoral Tregs, and altered the phenotype of Tregs into effector like T cells, and improved the response to ICIs (Fig. 5) [92].

##### EZH2 inhibitor promoted CD4^+^ and CD8^+^ T effector cells (Teffs) activity and trafficking to the TME

EZH2 expression increased after T cells activation or treating with ICIs [96], while EZH2 inhibitors combined with ICIs increased the expression of CXCL9 or CXCL10 [97] and the infiltration of Teffs [98, 99], enhanced the activity of CD4^+^ Teffs and CD8^+^ Teffs [92, 95], increased the production of INF-γ (Fig. 5) [92]. Furthermore, inhibition of EZH2 can increase PD-L1 expression by downregulating the H3K27me3 levels on the promoters of CD274 (PD-L1 encoding gene) [100], and upregulated PD-1 expression on CD8^+^ T infiltrating cells (Fig. 5) [99].

#### EZH2 and VEGF

It was demonstrated that EZH2 expression was positively correlated with VEGF-A expression and AKT phosphorylation, and EZH2 promoted tumor growth via the VEGF-A/AKT signaling pathway [101]. However, Riquelme [79] demonstrated that VEGF binding to VEGFR induced EZH2 expression through the upregulation of E2F3 and hypoxia-inducible factor-1α (HIF1α) [102].

In conclusion, EZH2 exerts suppressive effects on the antitumor effects of innate and adaptive immunity. However, EZH2 inhibitor (EZH2i) reverses the suppressive effects by modulating TME, and even enhances the efficacy of ICIs.

#### The role of EZH2 inhibitor

EZH2 inhibitor induced cell cycle arrest, apoptosis and differentiation of tumor cells, and reduced tumor size [103, 104].

Tazemetostat (a selective EZH2 inhibitor) exhibited potent anti-proliferation and antitumor effects in SCCOHT cell lines and xenografts which are both SMARCA2 and SMARCA4 deficient [82]. In preclinical research, JQEZ5, a EZH2 inhibitor, has shown anti-tumor activity in mice and human lung adenocarcinoma model of high EZH2 expression [105]. Moreover, GSK126, another EZH2 inhibitor, suppressed VEGF-A expression and AKT phosphorylation, and inhibited cell proliferation, migration and metastasis in lung cancer [101]. Tazemetostat showed a favourable safety profile and antitumor activity in patients with refractory B-cell non-Hodgkin lymphoma and advanced solid tumours [106]. In a phase 2 basket trial, the median duration of response was not reached (95% CI 9.2-not estimable), and the mOS was 19.0 months (11.0-not estimable), which indicated that Tazemetostat has the potential to improve outcomes in patients with advanced SWI/SNF-deficient solid tumor [107].

## Prognosis

SMARCA4-UT is extremely invasive with poor prognosis, and the mOS is 6 months [8]. The expression level of PD-L1 is positively correlated with the response to immunotherapy to some extent. For SMARCA4-UT patients with PD-L1 expression levels of 1% or more, the response to ICIs appears to be satisfactory. It seems that the higher the PD-L1 expression level, the better the efficacy of immunotherapy. However, the benefit from immunotherapy may not be satisfactory when high PD-L1 expression is accompanied with *KEAP1* mutation [20]. Moreover, the presence of TLS in tumor seems to be associated with improved prognosis [19].

## Conclusion

WHO has classified SMARCA4-UT as “other epithelial neoplasms of the lung” in 2021 to increase focus on SMARCA4-UT. If undifferentiated or poorly differentiated thorax tumors, especially for those are apparently local infiltration accompanied by distant metastasis, occur in young male patients with a heavy smoking history, it should be alert to SMARCA4-UT. Immunohistochemical staining for SMARCA4, SMARCA2, SOX2 and Claudin-4 should be performed in combination with NGS simultaneously.

Currently, there is no standard treatment regime for SMARCA4-UT. Patients with early or locally advanced disease may benefit from radical surgery and adjuvant chemotherapy (or combined with Bevacizumab concurrently). Reoperation, radiotherapy or oncolytic therapy may prolong the survival time of patients who had undergone surgery and locally progressed.

Compared with chemotherapy alone, the prognosis of patients with unresectable or inoperable SMARCA4-UT who undergo radiotherapy and platinum-based chemotherapy may be improved.

High PD-L1 expression level or the presence of TLS in tumors appears to be associated with better prognosis. For unresectable tumors or inoperable patients with PD-L1 expression of at least 1%, PD-1 inhibitor monotherapy (PD-L1 > 50%) or combined with platinum-based chemotherapy in the first-line seems to be associated with improved prognosis. Furthermore, patients without negative predictors of immunotherapy efficacy such as *KEAP1* mutation may benefit most from ABCP.

However, it is necessary to validate these inferences in preclinical and clinical practice.

Negative regulation on TME of EZH2 has been gradually revealed. Antitumor and immunomodulation of EZH2 inhibitors and their synergistic effects with ICIs provide a rationale for their combined utilization with ICIs or alone.

In recent years, new targeted therapies and epigenetic regulators including CDK4/6 inhibitor, OXPHOS inhibitor, BETi, AURKA inhibitor, PARP inhibitor, and ATR inhibitor are emerging. In particular, KRAS inhibitor, AXL inhibitor, and HDACi have obtained promising results, and they combined with PD-1 inhibitor may improve the prognosis of patients with SMARCA4-UT in the future.

Here, we propose that patients with SMARCA4-UT may benefit more from PD-1 inhibitor combined with platinum-based chemotherapy, anti-VEGF therapy combined with PD-L1 inhibitor plus chemotherapy. In addition, based on the pathogenesis, epigenetic characteristics, and co-mutations of SMARCA4-UT, PD-1 inhibitor combined with HDACi, KRAS inhibitor, AXL inhibitor or EZH2 inhibitor may be of great potential.

### Supplementary Information

Supplementary Information
