# Supplementary material for: The golden key to open mystery boxes of SMARCA4-deficient undifferentiated thoracic tumor: focusing immunotherapy, tumor microenvironment and epigenetic regulation
Source: Cancer Gene Ther. 2024 Feb 13;31(5):687–97. doi: 10.1038/s41417-024-00732-4 (PMC11101339; doi:10.1038/s41417-024-00732-4)
Supplement: Supplementary file 1 — Supplementary Information [file 41417_2024_732_MOESM1_ESM.docx]

| Patients | Gender/  Age  (years) | Size  (mm) | Main primary lesion  (At Presentation) | Metastatic Disease  (At Presentation) | TNM  Staging‡ | Treatment 1 | Outcome 1 | Interval Time 1 | Treatment 2 | Outcome 2 | Interval Time 2 | Treatment 3 | Outcome 3 | Interval Time 3 | Vital Status | PD-L1 | TMB  (Muts/Mb) | Driver Gene | Ref. |
| --- | --- | --- | --- | --- | --- | --- | --- | --- | --- | --- | --- | --- | --- | --- | --- | --- | --- | --- | --- |
| 1 | M/62 | 130*240 | Anterior mediastinum | — | IIIA  (T3N0M0) | S | Local relapse | 1 mo | Erlotinib | Left adrenal gland and CNS mets | 15 mo | NA | NA | NA | DOD  (20 mo) | NA | NA | None | 1 |
| 2 | M/61 | 35*30*  20 | Left lung apex | — | IB  (T2aN0M0) | S+AP+Avastin | Adrenal gland met | 12 w | DOC/Avastin | Adrenal gland progression | NA | NA | NA | NA | DOD  (16 mo) | NA | NA | None | 1 |
| 3 | F/28 | 130*120*150 | Anterior mediastinum | — | IIIB（T4N0M0） | nCT (VIDE)+S | NA | NA | NA | NA | NA | NA | NA | NA | DOD  (11 mo) | NA | NA | None | 1 |
| 4 | M/55 | 210*140*110 | Anterior mediastinum | — | IVA（T3N1M0） | nCT (EP)+S+RT | Local relapse | 1 mo post surgery | Lucitanib | Local progression | NA | NA | NA | NA | DOD  (7 mo) | NA | NA | None | 1 |
| 5 | M/59 | 50*30*  30 | RUL | — | IIA (T2bN0M0) | Palliative | Local progression | 5 w | RUL lobectomy and adjuvant CT | SD⁂ | 3 mo | — | — | — | Alive  (5.5 mo+) | 1% | NA | None | 22 |
| 6 | F/ early 30's | 78*41,  53*32 | Lung and mediastinum (2 separate masses) | — | IIIA (T4N0M0) | ADM | PD⁂ | 20 w | Surgery | NA | NA | NA | NA | NA | Lost | NA | NA | NA | 21 |
| 7 | M/72 | 91 | Right parietal pleura | — | IIIB（T4N2M0） | ADM | Local progression | 18 w | NA | NA | NA | NA | NA | NA | DOD  (9 mo) | NA | NA | None | 1 |
| 8 | M/57 | 140*120 | Superior and middle mediastinum | NA | IV  (T4N3M1x) | EP | Pleural progression | 18 w | PTX | PD | 3 w | NA | NA | NA | DOD  (8 mo) | NA | NA | None | 1 |
| 9 | M/50 | 90*62*  63 | Parietal pleura and right lung apex | NA | IVA (T4N1M1b) | RT+MDI | Local progression | 9 w | CTX/NVB | Local progression and CNS met | 9 w | NA | NA | NA | Alive  (12 mo+) | NA | NA | None | 1 |
| 10 | M/40 | 48 | Mediastinum | — | IV  (TxN1+M0) | Ipi/Nivo | NA | NA | NA | NA | NA | NA | NA | NA | DOD  (1.2 mo) | Ne | NA | NA | 19 |
| 11 | F/66 | NA | Mediastinum | Bone, adrenal glands | IVB  (TxN1+M1b) | Nivo | NA | NA | NA | NA | NA | NA | NA | NA | DOD  (2.2 mo) | Ne | NA | NA | 19 |
| 12 | M/69 | 42 | Lung | — | T2bNxM0 | TP | PD | NA | Nivo | PD | NA | NA | NA | NA | DOD  (6.5 mo) | Ne | NA | NA | 19 |
| 13 | F/58 | NA | Mediastinal, hilar and paratracheal | Pleura and peritoneum | IVB  (TxNxM1b) | CRT (TP) | PD | NA | Pembro | PR⁂ | — | — | — | — | Alive  (11 mo+) | Ne | NA | NA | 66 |
| 14 | M/76 | NA | Anterior mediastinal | NA | IVB  (T3N2Mx) | CRT (TP) | Bone mets | NA | Open fixation surgery and adjuvant RT(bone)+EC | Local progression and external iliac and inguinal lymph nodes mets | NA | Nivo | PR | — | Alive  (22 mo+) | <1% | 15 | None | 67 |
| 15 | M/45 | 50 | Lung | Left adrenal | IVA (T2bN0M1a) | TP⁋+ICI | PD | 24 w | Aminectomy for decompression of the spine and palliative | NA | NA | NA | NA | NA | DOD  (11 mo) | 0% | NA | None | 4 |
| 16 | M/58 | NA | Right pleura | Adrenal gland | IV  (TxNxM1) | AP+Pembro (3 cycles), Pembro | PR  (after intial therapy 3 mo) | 3 mo+ | — | — | — | — | — | — | Alive  (11 mo+) | 10% | NA | None | 68 |
| 17 | M/41 | 120*100*57 | Superior and anterior mediastinum | — | IVB  (T3N2M0) | Pembro | Cervical and mediastinal lymph nodes mets | 24 w | Ipi+ Pembro | Supraclavicular lymph nodes mets | 12 w | TP, RT after 5mo, and CRT(ADM/ IFO), successively | PD | 6w, NA and NA, successively | DOD  (26 mo) | 100% | High | None | 69 |
| 18 | F/69 | NA | Left mediastinum | Peritoneum, retroperitoneum, skin | IVB  (TxN0M1b) | Pembro | PR  (1 cycle) | 24 w+ | — | — | — | — | — | — | Alive  (6 mo+) | >60% | None | None | 70 |
| 19 | M/59 | 64 | LLL | Oropharynx, left adrenal gland and abdominal lymphadenopathies | IVB (T3N3M1c) | ABCP (3 cycles) and AB⁑ | PD | 10 mo | — | — | — | — | — | — | Lost | 0 | 11.8 | None | 20 |
| 20 | F /73 | 45 | LUL | Bone | IVA (T2bN2+M1b) | ABCP (3 cycles) and AB⁑ | PR  (3 cycles) | 17 mo+ | — | — | — | — | — | — | Alive  (17 mo+) | 40% | 11 | None | 20 |
| 21 | F/64 | 27 | LUL | Brain | IVA  (T1cN2+M1b) | ABCP | Brain met | 12 w | RT(Brain)+AB | PD | 2 mo | — | — | — | Lost | 80% | 14.9 | None**∮** | 20 |
| 22 | M/39 | NA | Lung | Jaw | IVA (TxN0M1b) | Surgery(jaw met) and adjuvant CT(ADM/IFO and ACTD/ DTIC/DDP, successively) | PD | NA | Ipi+Nivo, surgery(residual lung mass) and adjuvant Nivo | CR⁂ | — | — | — | — | Alive (almost 2 years) | >1% | 29 | NA | 19 |

**‡ According to main primary lesion, using IASLC Eighth Edition of the TNM Classification for Lung Cancer, Thymic Epithelial Tumors, or Pleural Mesothelioma to re-stage the cases.**

**⁋ nab-Paclitaxel/Carboplatin**

**⁂ Using RECIST version 1.1 or iRECIST to evaluate disease response**

**⁑ Due to adverse effects, AB maintenance therapy was given after 3 cycles of ABCP.**

**∮***KEAP1* mutation

**Abbreviations: ABCP: Atezolizumab/Bevacizumab/Carboplatin/Paclitaxel; ACTD: Actinomycin D; AP: Permetrexed/Carboplatin; ADM: Doxorubicin; CNS: Central Nervous System; CR: Complete Response; CRT : Chemotherapy+Radiotherapy; CT : Chemotherapy; CTX/NVB: Cyclophosphamide/Vinorelbine; DTIC: Dacarbazine; DDP: Cisplatin; DOC: Docetaxel; DOD: Death of Disease; EC: Etoposide/Carboplatin; EP: Etoposide/Cisplatin; F: female; ICI: Immune Checkpoint Inhibitor; IFO: Ifosfamide; Ipi: Ipilimumab; LLL: Left Lower Lobe; Lost: Lost follow-up; LUL: left upper**

**lobe; M: male; MDI: Mesna/Doxorubicin/Ifosfamide; met: metastasis; mo: months; NA: not available; Ne: Negative; Nivo: Nivolumab; nCT : Neoadjuvant Chemotherapy; OS: Overall Survival;** Ref.: References; **PD: Progressive Disease; Pembro: Pembrolizumab; PR: Partial Response; PTX: Paclitaxel; RT : Radiotherapy; RUL: right upper lobe; S: Radical surgery; SD: Stable Disease; TP: Paclitaxel/Carboplatin; VIDE: Vincristine/Ifosfamide/Doxorubicin/Etoposide; w: weeks**
